# Supplementary material for: The Multi-Sites Trial on the Effects of Therapeutic Gardening on Mental Health and Well-Being
Source: Int J Environ Res Public Health. 2022 Jun 30;19(13):8046. doi: 10.3390/ijerph19138046 (PMC9266122; doi:10.3390/ijerph19138046)
Supplement: Supplementary file 1 [file ijerph-19-08046-s001.zip › ijerph-1775572-supplementary.pdf]

Table S1. The example of therapeutic gardening program.

Session	Activity	Session	Activity
1	Introduction	16	Harvesting vegetables
2	Preparing gardening	17	Setting up autumn garden
3	Setting up kitchen garden	18	Making terrarium
4	Setting up seasonal bed	19	Walking in a garden
5	Setting up herb bed	20	Propagating plants with suckering
6	Health lecture	21	Propagating plants with stem cutting
7	Setting up aquatic garden	22	Setting up grass garden
8	Setting up rock garden	23	Plant Pruning
9	Sowing, mulching, composting	24	Making autumn wreath
10	Making herbal soap	25	Walking in a garden
11	Walking in a garden	26	Collecting seed
12	Irrigation, removing weed	27	Making bird house
13	Pest control	28	Making own mini garden
14	Setting up butterfly garden	29	Planting fall bulbs
15	Setting up hanging garden	30	Garden party, exhibition

Table S2. The result of moderation analysis of gender.

	Effects Estimate	SE	df	t	*p*
**MHS:D**					
Intercept	12.067	0.885	637	13.630	< 0.0001
Time	-1.144	0.151	637	-7.568	< 0.0001
Gender	2.332	1.961	109	1.190	0.237
Time*Gender	0.686	0.338	637	2.031	0.043*
**MHS:A**					
Intercept	11.233	0.904	638	12.427	< 0.0001
Time	-0.855	0.134	638	-6.396	< 0.0001
Gender	2.537	2.001	109	1.268	0.208
Time*Gender	0.370	0.300	638	1.232	0.218
**EDAS**					
Intercept	16.908	0.363	637	46.573	< 0.0001
Time	0.239	0.065	637	3.697	0.0002
Gender	-0.166	0.806	109	-0.206	0.837
Time*Gender	0.065	0.145	637	0.449	0.653
**WHOQOL -BREF**					
Intercept	81.948	1.539	96	53.250	< 0.0001
Time	9.800	1.841	90	5.322	< 0.0001
Gender	-0.859	4.198	96	-0.205	0.838
Time*Gender	7.949	5.284	90	1.504	0.136
**MAAS**					
Intercept	60.272	1.419	102	42.464	< 0.0001
Time	6.401	1.795	97	3.566	0.0006
Gender	-5.512	3.264	102	-1.689	0.094
Time*Gender	1.318	4.178	97	0.315	0.753

Note: SE = Standard Error. df = degree of freedom. t = t-value. * p-value < 0.05. MHS:D—Mental Health Screening Tool for Depressive disorders; MHS:A—Mental Health Screening Tool for Anxiety disorders; EDAS—Engagement in Daily Activity Scale; WHOQOL-BREF—Brief version of WHO Quality of Life; MAAS—Mindful Attention Awareness Scale.

Table S3. The result of moderation analysis of age.

	Effects Estimate	SE	df	t	*p*
**MHS:D**					
Intercept	10.607	1.267	637	8.374	< 0.0001
Time	-1.495	0.216	637	-6.917	< 0.0001
Age	3.151	1.613	109	1.953	0.053
Time*Age	0.787	0.274	637	2.870	0.0042*
**MHS:A**					
Intercept	8.481	1.255	638	6.759	< 0.0001
Time	-0.782	0.197	638	-3.960	0.0001
Age	5.289	1.597	109	3.312	0.001
Time*Age	0.007	0.250	638	0.029	0.977
**EDAS**					
Intercept	17.925	0.506	637	35.399	< 0.0001
Time	0.326	0.094	637	3.472	0.0006
Age	-1.709	0.644	109	-2.625	0.009
Time*Age	-0.123	0.119	637	-1.029	0.304
**WHOQOL -BREF**					
Intercept	84.031	2.226	96	37.746	< 0.0001
Time	10.213	2.711	90	3.767	0.0003
Age	-3.852	2.898	96	-1.329	0.187
Time*Age	0.974	3.538	90	0.275	0.784
**MAAS**					
Intercept	61.425	2.160	102	28.442	< 0.0001
Time	7.391	2.721	97	2.717	0.008
Age	-3.415	2.689	102	-1.270	0.207
Time*Age	-1.101	3.385	97	-0.325	0.746

Note: SE = Standard Error. df = degree of freedom. t = t-value. * p-value < 0.05. MHS:D—Mental Health Screening Tool for Depressive disorders; MHS:A—Mental Health Screening Tool for Anxiety disorders; EDAS—Engagement in Daily Activity Scale; WHOQOL-BREF—Brief version of WHO Quality of Life; MAAS—Mindful Attention Awareness Scale.

Table S4. The result of moderation analysis of presence of mental disorder.

	Effects Estimate	SE	df	t	*p*
**MHS:D**					
Intercept	8.523	1.027	637	8.297	< 0.0001
Time	-1.265	0.196	637	-6.445	< 0.0001
MD	7.626	1.415	109	5.391	< 0.0001
Time*MD	0.502	0.273	637	1.841	0.0661*
**MHS:A**					
Intercept	8.555	1.106	638	7.737	< 0.0001
Time	-0.978	0.172	638	-5.696	< 0.0001
MD	6.055	1.522	109	3.978	0.0001
Time*MD	0.383	0.240	638	1.599	0.110
**EDAS**					
Intercept	16.342	0.466	637	35.041	< 0.0001
Time	0.324	0.083	637	3.920	0.0001
MD	1.002	0.642	109	1.559	0.122
Time*MD	-1.139	0.115	637	-1.205	0.229
**WHOQOL-BREF**					
Intercept	83.306	1.942	96	42.897	< 0.0001
Time	15.882	2.205	90	7.202	< 0.0001
MD	-3.582	2.857	96	-1.254	0.213
Time*MD	-11.390	3.295	90	-3.456	0.0008*
**MAAS**					
Intercept	62.586	1.848	102	33.869	< 0.0001
Time	8.922	2.334	97	3.822	0.0002
MD	-6.324	2.527	102	-2.502	0.014
Time*MD	-4.326	3.209	97	-1.348	0.181

Note: SE = Standard Error. df = degree of freedom. t = t-value. * p-value < 0.05. MHS:D—Mental Health Screening Tool for Depressive disorders; MHS:A—Mental Health Screening Tool for Anxiety disorders; EDAS—Engagement in Daily Activity Scale; WHOQOL-BREF—Brief version of WHO Quality of Life; MAAS—Mindful Attention Awareness Scale.

Table S5. The result of multilevel analysis of each mental disorder for 'Mental Health Screening Tool for Depressive disorders'.

	Effects Estimate	SE	df	t	*p*
**Depressive Disorder**					
Intercept	16.474	2.482	86	6.639	< 0.0001
Time	-1.084	0.486	86	-2.232	0.0282*
**Schizophrenia**					
Intercept	16.682	2.679	77	6.227	< 0.0001
Time	-0.120	0.593	77	-0.202	0.8406
**Neurocognitive Disorder**					
Intercept	13.051	1.263	49	10.333	< 0.0001
Time	-1.450	0.273	49	-5.311	< 0.0001*
**Neurodevelopmental Disorder**					
Intercept	16.463	3.396	68	4.848	< 0.0001
Time	-0.216	0.456	68	-0.474	0.6368
**Anxiety Disorder**					
Intercept	32.377	5.625	6	5.756	0.0012
Time	-2.076	1.114	6	-1.864	0.1116
**Bipolar Spectrum Disorder**					
Intercept	21.970	4.131	24	5.319	< 0.0001
Time	-0.496	0.968	24	-0.513	0.6127

Note. SE = Standard Error. df = degree of freedom. t = t-value. * *p*-value < 0.05.

Table S6. The result of multilevel analysis of each mental disorder for 'Mental Health Screening Tool for Anxiety disorders'.

	Effects Estimate	SE	df	t	*p*
**Depressive Disorder**					
Intercept	15.685	2.302	86	6.81	< 0.0001
Time	-1.220	0.299	86	-4.08	0.0002*
**Schizophrenia**					
Intercept	16.147	2.496	77	6.469	< 0.0001
Time	-0.578	0.509	77	-1.134	0.2603
**Neurocognitive Disorder**					
Intercept	8.606	2.599	49	3.311	0.0017
Time	-0.017	0.170	49	-0.100	0.9209
**Neurodevelopmental Disorder**					
Intercept	15.644	3.101	68	5.045	< 0.0001
Time	-0.533	0.521	68	-1.022	0.3106
**Anxiety Disorder**					
Intercept	16.944	4.102	6	4.130	0.0061
Time	0.358	1.790	6	0.200	0.8481
**Bipolar Spectrum Disorder**					
Intercept	22.762	3.451	24	6.595	< 0.0001
Time	-0.411	1.029	24	-0.400	0.6928

Note. SE = Standard Error. df = degree of freedom. t = t-value. * *p*-value < 0.05.

Table S7. The result of multilevel analysis of each mental disorder for 'Engagement in Daily Activity Scale'.

	Effects Estimate	SE	df	t	*p*
**Depressive Disorder**					
Intercept	14.601	0.647	86	22.556	< 0.0001
Time	0.182	0.167	86	1.089	0.2794
**Schizophrenia**					
Intercept	16.717	0.963	77	17.358	< 0.0001
Time	0.252	0.148	77	1.697	0.0937
**Neurocognitive Disorder**					
Intercept	20.642	0.938	49	22.002	< 0.0001
Time	0.317	0.198	49	1.602	0.1156
**Neurodevelopmental Disorder**					
Intercept	18.257	1.169	68	15.619	< 0.0001
Time	0.022	0.197	68	0.112	0.9108
**Anxiety Disorder**					
Intercept	10.750	1.734	6	6.201	0.0008
Time	0.429	0.757	6	0.566	0.5916
**Bipolar Spectrum Disorder**					
Intercept	15.055	0.981	24	15.3	0.0012
Time	-0.104	0.174	24	-0.6	0.5714

Note. SE = Standard Error. df = degree of freedom. t = t-value.

Table S8. The result of multilevel analysis of each mental disorder for 'Brief version of WHO Quality of Life'.

	Effects Estimate	SE	df	t	*p*
**Depressive Disorder**					
Intercept	70.318	4.001	10	17.574	< 0.0001
Time	8.273	3.894	10	2.124	0.060
**Schizophrenia**					
Intercept	72.125	9.159	3	7.875	0.0043
Time	5.750	6.343	3	0.907	0.4315
**Neurocognitive Disorder**					
Intercept	85.143	2.528	13	33.686	< 0.0001
Time	2.000	4.728	13	0.423	0.6792
**Neurodevelopmental Disorder**					
Intercept	88.498	5.408	12	16.365	< 0.0001
Time	2.689	5.467	8	0.492	0.636
**Bipolar Spectrum Disorder**					
Intercept	64.177	4.390	2	14.620	0.0046
Time	-4.313	8.755	1	-0.493	0.7086

Note. Anxiety disorder cannot be analyzed due to insufficient data (N = 1). SE = Standard Error. df = degree of freedom. t = t-value.

Table S9. The result of multilevel analysis of each mental disorder for 'Mindful Attention Awareness Scale'.

	Effects Estimate	SE	df	t	*p*
**Depressive Disorder**					
Intercept	57.000	3.208	12	17.771	< 0.0001
Time	2.154	5.545	12	0.389	0.7045
**Schizophrenia**					
Intercept	50.083	4.320	11	11.595	< 0.0001
Time	2.167	3.191	11	0.679	0.5111
**Neurocognitive Disorder**					
Intercept	60.250	4.255	11	14.159	< 0.0001
Time	8.333	6.117	11	1.362	0.2003
**Neurodevelopmental Disorder**					
Intercept	58.110	4.047	13	14.358	< 0.0001
Time	6.507	3.837	10	1.696	0.1208
**Bipolar Spectrum Disorder**					
Intercept	49.861	3.475	3	14.348	0.0007
Time	1.223	6.029	2	0.203	0.8580

Note. Anxiety disorder cannot be analyzed due to insufficient data (N = 1). SE = Standard Error. df = degree of freedom. t = t-value.
